# Benchmarking of a Simple Scintigraphic Test for Gastro-oesophageal Reflux Disease That Assesses Oesophageal Disease and Its Pulmonary Complications

**DOI:** 10.4274/mirt.10438

**Published:** 2018-10-09

**Authors:** Leticia Burton, Gregory L. Falk, Stephen Parsons, Mel Cusi, Hans Van Der Wall

**Affiliations:** 1 CNI Molecular Imaging, Sydney, Australia; 2 Sydney Heartburn Clinic, Sydney, Australia; 3 University of New South Wales, Sydney, Australia; 4 University of Notre Dame, Sydney, Australia

**Keywords:** Gastro-esophageal, reflux, scintigraphy, manometry, aspiration, pulmonary

## Abstract

**Objectives:**

Gastro-oesophageal reflux disease (GORD) is both common and troubling with a prevalence of 20-40%. We assessed the utility of a scintigraphic reflux study to evaluate the oesophageal and extra-oesophageal manifestation of disease compared to the standard tests such as pH monitoring and manometry.

**Methods:**

Patients were recruited into a prospective database of referrals to a tertiary referral center for either resistance to maximal medical therapy or extra-oesophageal symptoms of GORD. Data included 2 channel 24-hour pH monitoring and manometry results, as well as scintigraphic reflux data with late images assessing pulmonary aspiration of refluxate.

**Results:**

Study population included 250 patients (155 F, 95 M) with an average age of 60 years. Patients were clinically classified as either GORD (n=72) or laryngopharyngeal reflux (LPR) (n=178). Pulmonary aspiration of the refluxate was detected significantly more commonly in LPR patients (58/178 compared with GORD 10/72). Strong correlations were found between the scintigraphic time-activity curves in the upper oesophagus and pharynx, and ineffective oesophageal motility and pulmonary aspiration. pH studies correlated with the scintigraphic studies but did not predict aspiration similar to other modalities when evaluated by ROC analysis.

**Conclusion:**

Scintigraphic reflux studies offer a viable alternative test for GORD and extra-oesophageal manifestations of reflux disease. Strong correlations were found between measurable scintigraphic parameters and oesophageal motility and lung aspiration of refluxate. This may provide a more confident decision analysis in patients being considered for fundoplication for troubling extra-oesophageal symptoms.

## Abstract (tr)

**Amaç:**

Gastro-özofageal reflü (GÖR) hastalığı %20-40 arası prevalansı ile sık görülen bir sorundur. Bu çalışmada hastalığın özofageal ve özofagus dışı belirtilerini değerlendirmek için sintigrafik reflü testinin yararını inceleyerek bunu pH monitorizasyonu ve manometri gibi standart testlerle karşılaştırdık.

**Yöntem:**

Maksimal medikal tedavi veya GÖR özofagus-dışı belirtiler nedeniyle üçüncü basamak bir referans merkezine yönlendirilmiş hastalar prospektif veri tabanında toplandı. İki kanal 24-saat pH monitorizasyonu ve manometri sonuçları ile reflüksatın pulmoner aspirasyonunu değerlendiren geç görüntülerin dahil edildiği sintigrafik reflü verileri saptandı.

**Bulgular:**

Çalışmaya ortalama 60 yaşında 250 hasta (155 K, 95 E) dahil edildi. Hastalar klinik olarak ya GÖR (n=72) ya da laringofaringeal reflü (LPR) (n=178) olarak ikiye ayrıldı. Reflüksatın pulmoner aspirasyonu LPR hastalarında GÖR hastalarına göre anlamlı olarak daha sık saptandı (58/178’e vs. 10/72). Üst özofagus ile farinkste sintigrafik zaman-aktivite eğrileri arasında ve inefektif özofageal motilite ve pulmoner aspirasyon arasında ciddi korelasyon saptandı. pH testleri sintigrafik incelemelerle uyumlu idi ancak diğer incelemeler gibi ROC analizi ile değerlendirildiğinde sintigrafi de aspirasyonu öngöremedi.

**Sonuç:**

Sintigrafik reflü incelemeleri GÖR ve reflü hastalığının özofagus dışı belirtileri için yararlı bir alternatif testtir. Ölçülebilir sintigrafik parametreler ile özofagus motilitesi ve reflüksatın akciğer aspirasyonu arasında ciddi korelasyon saptanmıştır. Bu inceleme, özofagus dışı semptomu olan ve fundoplikasyon için değerlendirilen hastalarda ilgili karar verme sürecine katkı sağlayabilir.

## Introduction

Gastro-oesophageal reflux disease (GORD) is a common and troubling problem that has a prevalence of 20-40% in its various complex manifestations (1). Variability depends on the criteria utilized in the definition and has now been expanded to “a condition that develops when the reflux of stomach contents causes troublesome symptoms and/or complications” (Montreal definition, 2006) (2). A problem with the definition is that it encompasses many non-specific symptoms and requires confirmation by endoscopy or the application of a therapeutic trial with a clinical response to confirm the diagnosis. Endoscopy is necessary to confirm the presence of esophagitis and exclude sinister pathology. Even so, endoscopy will miss a high proportion of uncomplicated GORD (>50%) and between 25% and 40% of patients will remain unresponsive or refractory to such clinical trials (3).

These circumstances have led to the requirement for invasive testing such as 24-hour pH monitoring, manometry and impedance reflux measurements. Such testing will fundamentally assess the presence of acidic reflux, lower oesophageal sphincter (LOS) pressures/oesophageal clearance or non-acid reflux, respectively (4). However, the blind spot of these tests is in assessment of the extra-esophageal manifestations of reflux such as cough, recurrent sinusitis, laryngitis or chronic recurrent chest symptoms, particularly in those subgroups who experience silent (or non-heartburn) GORD. Laryngopharyngeal reflux (LPR) and lung aspiration of refluxate are dangerous complications that often occur in the absence of oesophagitis or its primary symptom, heartburn (5). LPR complications have been reported in numerous publications and have been succinctly summarized by Koufman et al (5). These complications include laryngeal carcinoma, vocal cord nodules, laryngospasm and subglottic stenosis.

While there have been a number of scintigraphic reflux studies in the past (6,7,8), there has been no general acceptance of the technique due to the variability in technique and inconsistent results. We have validated (9) and present a simple modification of the existing scintigraphic reflux testing and benchmark the findings against the current reference standards such as 24-hour pH monitoring and manometry. The comparison with impedance will be reported separately. We hypothesized that scintigraphic reflux testing is capable of assessing both the presence of oesophageal disease and its extra-oesophageal manifestations with good correlation with existing testing regimens.

## Materials and Methods

### Population and Clinical Data

A database of patients with either proven or suspected GORD/LPR [approved by the Institutional Ethics Committee (LNR/12 CRGH/248)] was maintained prospectively. Patients being investigated for suspected GORD/LPR disease with pH/manometry studies was extracted from the database. Patients were chosen for the study if they had mainly upper respiratory tract symptoms that remained undiagnosed after 8 weeks of investigation by appropriate specialists and classified according to the reflux symptom index criteria of Belafsky et al (10). Major upper respiratory tract symptoms documented included cough, sore throat, recurrent throat clearing, voice change, laryngospasm, aspiration, globus and regurgitation. A history of heartburn, regurgitation and dysphagia was routinely elicited. All patients had severe symptoms that were resistant to high-dose proton-pump inhibitor therapy and had been referred for consideration of fundoplication. Scintigraphy was used to prospectively evaluate extra-oesophageal refluxate and the possibility of pulmonary aspiration of refluxate. This is therefore a highly selected group of patients with a strong pre-test probability of GORD causing LPR. A large proportion had a long history of undiagnosed upper respiratory tract symptoms and were studied by scintigraphy in order to evaluate the possibility of reflux disease as a causation. Clinical data was prospectively collected using a standardized proforma and entered into a database.

### pH Monitoring (2 Channel)

24-hour impedance reflux study with two channel 24-hour pH was performed on all patients. Following local anesthetic application, a trans-nasal catheter was introduced into the oesophagus. This consisted of 2 level impedance rings and 2 level pH electrodes connected to an external monitoring device and calibrated accordingly. Impedance rings were maneuvered to 5 and 15 cm above the upper border of the LOS (Zephyr device, catheter ZAI-BD31, Sandhill Co, Highlands Ranch, Colorado, USA). No dietary restrictions were made other than ingestion of acidic beverages. Catheter placement was checked by manometry with the lower pH electrode 5 cm above the upper border of the LOS and the upper, 15 mm higher. Patients returned the following day for removal of the assembly. Meal-times were included in the reporting analysis. Reports of 24-hour pH and 24-hour impedance reflux were generated by autoscan and manual review. Reflux was classified according to the consensus on impedance and pH monitoring (11). In summary, this is based on oesophageal pH during reflux detected by impedance monitoring. Acid reflux is defined as a fall in pH below 4, weakly acid reflux as a fall in pH which is ≥4 but <7 and non-acid reflux where oesophageal pH increases ≥7 or remains ≥7 during reflux.

### Manometry

Stationary manometry was obtained with a water perfused dent sleeve 8 channel catheter (Dent Sleeve International Mississauga, Ontario, Canada) using standard techniques. Data was recorded with a multichannel recording system (PC polygraph HR Medtronics, Synectics Medical, Minneapolis, Minnesota, United States) and analyzed using the PolyGram software program (Medtronics, Synectics Medical, Minneapolis, Minnesota, United States). Oesophageal motility was graded by the modified method of Kahrilas et al. (11,12). Grades were reported as normal, mildly, moderately or severely ineffective oesophageal motility (IOM). LOS pressure was recorded in all patients.

### Scintigraphy

Patients were fasted overnight and medications were ceased for 24 h prior to the test. Patients were positioned upright in front of a Hawkeye 4 gamma camera (General Electric, Milwaukee, United States) with the mandible and stomach in the field of view. They swallowed 40-60 MBq of Tc-99m DTPA diluted in 50 mL of water followed by an additional 50 mL of water to clear activity from the oropharynx and oesophagus.

**Figure 1:**
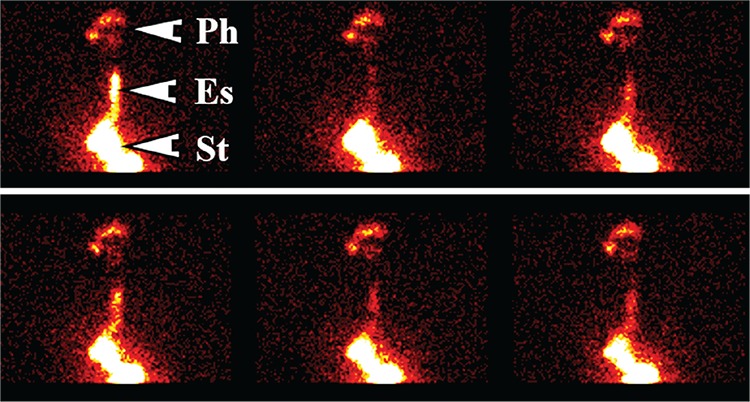
Dynamic sequence of the scintigraphic study showing full-column gastro-oesophageal reflux to the level of the pharynx. The oesophagus and stomach are labelled as Es and St, respectively

**Figure 2:**
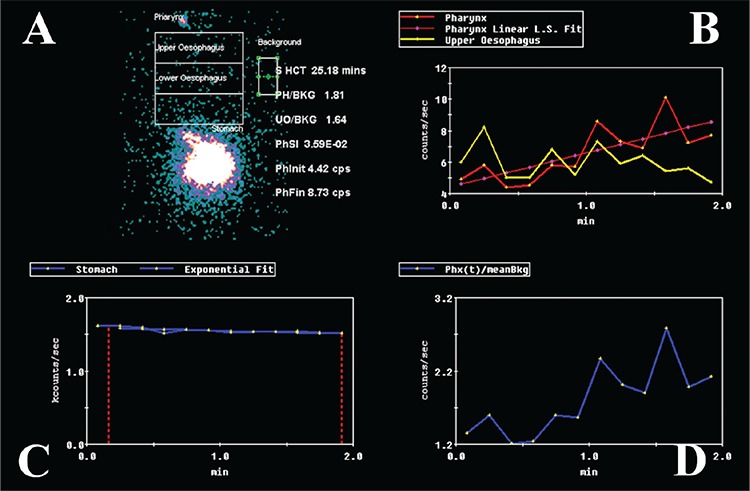
Graphical analysis of the dynamic study. Panel A shows the regions of interest for the pharynx, upper and lower oesophagus and the background regions as well as the relevant results. Panel B shows the time-activity curves for the pharynx (red) with its fitted curve (pink) and the curve for the upper oesophagus (yellow). Panel C is the gastric emptying curve with the time to half clearance being shown at 25.2 minutes in panel A. Panel D indicates the ratio of pharynx to background

**Figure 3:**
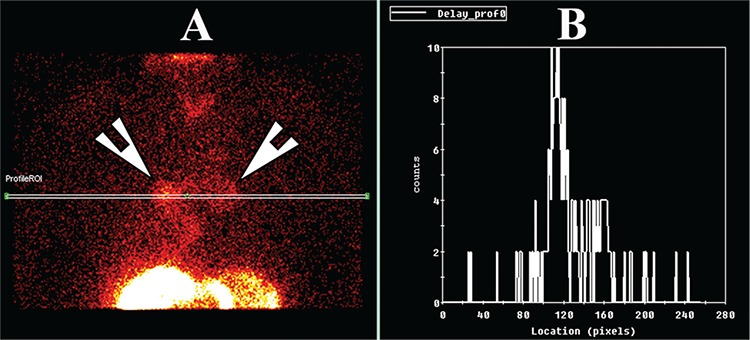
A) The delayed study at 2 hours demonstrates aspiration of tracer into both lungs with significant activity in the main airways (arrowheads). B) The line profile through the hilar regions shows the count densities in the lungs, which is 5 times higher than background activity

Dynamic imaging was performed for a duration of 2 minutes at an interval of 15s per frame into a 64*64 matrix. Patients were then placed in a supine position and dynamic images obtained at a framing rate of 30s per frame for 30 minutes. After supine imaging, 40-60 MBq of phytate (colloid) was administered orally with a 50 mL flush of water. Delayed static imaging using a 256*256 matrix was obtained two hours later for assessment of lung aspiration of refluxate. Images were analyzed by regions of interest over the pharynx, upper, mid and lower oesophagus with a background region over the lateral chest. Time-activity curves were generated from each region (Figure 1) Grades was assigned to the time-activity curves as shown in Figure 2. Grade 1 was a declining curve, with grade 2 being flat and grade 3 a rising curve. Delayed images (Figure 3) were analyzed with a line profile for the assessment of aspiration into the main airways (>2 X background).

### Statistical Analysis

Data was analyzed by nonparametric statistical methods as much of the analysis was of ordinal data with multiple studies for each patient. Standard ANOVA statistics, Wilcoxon matched pairs test, Student’s t-test and Pearson correlation coefficient (2 tails) with significance levels of 0.05 were utilized. Fisher’s exact test (two-tailed) and receiver operating characteristic (ROC) analysis was also undertaken where appropriate. Statistica V8 software (Statsoft, Oklahoma, United States) package was used for data analysis.

## Results

Population and clinical data. A total of 250 consecutive patients with complete data were studied (155 F, 95 M) over a period of 24 months. The average age was 60 years with a range of 20-85 years. Clinical history distinguished the patients clinically as predominantly GORD in 72 patients and LPR (±GORD) in 178. All patients underwent 24-hour pH monitoring and water perfused manometry. Scintigraphic studies were acquired within a 3-week period of the standard tests in all patients. A subset of 33 patients underwent laparoscopic fundoplication and these results have been reported elsewhere (9).

Two channel 24-hour pH monitoring. Twenty-four-hour pH studies were normal in 25 patients (pH >4), weakly acidic in 78 (pH >4, <7) and abnormal in the rest (147). Results of the pH findings are shown in Table 1. In patients with scintigraphic evidence of aspiration, 14% (n=10) had normal proximal pH studies while 6% (n=5) had normal distal pH studies.

There was no significant difference in pH studies between patients with LPR and GORD (p>0.05). Moderate correlation was found between proximal and distal acid exposure (Pearson correlation coefficient=0.32, p=0.001).

Proximal and distal acid exposure had no significant correlation with either LOS pressure or oesophageal clearance by manometry (p>0.05). Correlation coefficients were poor (Pearson correlation coefficients ranging from 0.080 to-0.15).

No significant correlation was found between proximal and distal acid exposure and either scintgraphic clearance curves from the pharynx or upper oesophagus (p>0.05).

**Manometry: **The patients clinically classified as LPR, had severe IOM (35%); compared to the GORD group (17%). This was a significant difference by Fisher’s exact test (two-tailed) with p=0.0058. Normal oesophageal motility was found in 27% with LPR symptoms and in 49% with GORD symptoms. This was a significant difference by Fisher’s exact test (two-tailed) with p=0.0021.

The mean LOS pressure was 6.3 mmHg [median: 2.3, standard deviation: 8.4 (95% CI: 5.9-7.6) mmHg]. No significant difference was found between the LPR and GORD groups for mean LOS pressure (p>0.01).

Severe IOM was strongly associated with isotope aspiration in both groups [p=0.00 for LPR (Pearson correlation coefficients: 0.54] and p=0.04 for GORD (Pearson correlation coefficients: 0.21).

There was a strong correlation between IOM and rising isotope curves in the pharynx when supine (Pearson correlation coefficients: 0.29, p=0.003) and upright (Pearson correlation coefficients: 0.38, p=0.00).

**Scintigraphy:** A total of 68 out of 250 patients demonstrated isotope aspiration into the lungs. There was significantly more pulmonary aspiration of refluxate in the group with LPR (58/178) symptoms than with a GORD profile (10/72) by Fisher’s exact test (p=0.0027).

The time activity curves for the pharynx and upper oesophagus with the pulmonary aspiration data for each pattern of curve is shown in Table 2 and 3, respectively. The outstanding feature of these findings is the rarity of isotope aspiration in patients with a declining time activity curve (Grade 1) for the pharynx and upper oesophagus. No patient with a clinical GORD profile had lung aspiration in either the upright or supine position and only 3 of 63 patients with LPR symptoms showed evidence of aspiration. Similar findings were shown for declining time-activity curves for the upper oesophagus. This is in sharp contrast with a rising time activity curve, where a high proportion of patients had evidence of pulmonary aspiration. A declining time-activity curve in the pharynx and upper oesophagus has a negative predictive value (NPV) of 97% for aspiration. Rising curves at both sites have a positive predictive value (PPV) of 98% for aspiration. The results for the pharynx, regardless of the upper oesophageal clearance pattern, were NPV of 98% and PPV of 100%.

**Figure 4:**
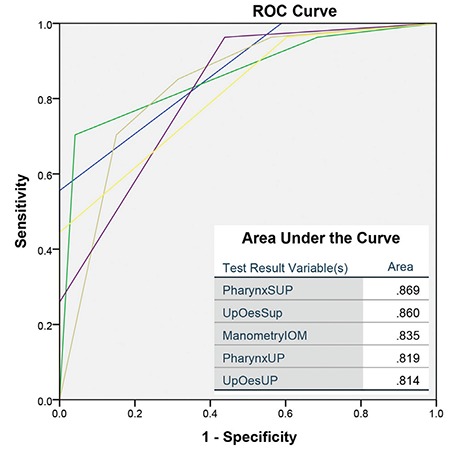
Receiver operating characteristic for the variables as predictors of lung aspiration of refluxate. The area under the curves is inset ROC: Receiver operating characteristic

The ROC analysis demonstrated that the optimal tests for pulmonary aspiration of refluxate were the scintigraphic time activity curves for the pharynx and upper oesophagus, and the manometric marker of oseophageal clearance (Figure 4). Distal oesophageal total acid exposure and LOS pressures were not significant predictors of lung aspiration (p>0.05) with proximal total acid exposure just reaching significance (p=0.04).

## Discussion

This study indicates that scintigraphic reflux studies are a viable alternative to the current suite of testing for the establishment of a diagnosis of GORD. However, the group of patients enrolled in the current study are not a typical representation of how this disease presents in the general community. This is a highly selected group of patients, referred to a tertiary center for resistance to standard therapy or atypical symptoms of GORD. Perhaps the most important finding of this study is that attempting to clinically classify patients as either purely oesophageal disease (GORD) or extra oesophageal disease (LPR) is a futile exercise. A significant proportion of patients classified as GORD will demonstrate pulmonary aspiration of refluxate, which is clinically silent (Figure 3). This has been elegantly shown by similar scintigraphic techniques in 20% of patients with chronic respiratory disease but silent GORD. As little as 0.1 MBq of aspirated activity was detectable in the lungs of these patients (13).

While the scintigraphic reflux study is capable of demonstrating evidence of GORD at the oesophageal level (Figure 1), its other great advantage is the delineation of extra-oesophageal disease. This is clearly reflected at the level of the oropharynx, laryngopharynx and the lungs. These areas are not screened by the existing suite of testing such as manometry and pH and with some reservations by impedance monitoring. Refluxate contaminating the extra-oesophageal tissues can be visualized and although 27% of patients showed evidence of pulmonary aspiration of refluxate, this may in fact be an underestimate of the true incidence of pulmonary aspiration in this type of patient cohort. Patients are supine for approximately 30 minutes and are essentially upright for the other 90 minutes prior to the delayed scan for pulmonary aspiration. This may in fact be significantly worse when the patient is supine and asleep at night (14).

Analysis of the scintigraphic time-activity curves for the pharynx and upper oesophagus showed a strong correlation with IOM, indicating that inability to adequately clear refluxate from the oesophagus is of significant importance in addition to the incompetence of the LOS tone in both GORD and LPR patients with pulmonary aspiration of refluxate. LOS tone was not a good discriminator as the majority of referred patients had poor tone with a mean of 6.3 mmHg (N~26 mmHg) (15). When analyzing the ROC curves, IOM was as useful as the scintigraphic time-activity curves in predicting aspiration of refluxate (Figure 4). This observation confirms that the scintigraphic technique is also useful in detecting dysmotility, as the time-activity curves will accurately reflect this. A rising curve is the end result of recurrent episodes of reflux and the inability of the oesophageal clearance mechanisms to remove the refluxate. Dysmotility is a key marker for LPR as has been shown by others, particularly in those with silent reflux and extra-oesophageal symptoms such as cough (16,17).

Earlier studies with 24-hour ambulatory pH monitoring have pointed erroneously out the importance of distal rather than proximal oesophageal pH as being important in patients with heart-burn and respiratory complications of GORD (18). Others have attempted to rationalize the disparity by suggesting that acid is neutralized during the ascent to the proximal oesophagus and may not register on the proximal pH probe (19). It is our contention that distal oesophageal pH does not fully emulate what is happening in the upper oesophagus and pharynx which is essentially beyond the level of the pH probe and therefore, is fundamentally a blind spot. This verifies the hypothesis of a poor correlation between pH studies in the distal oesophagus and lung aspiration of isotope to be true. The ROC analysis shows a poor performance for total distal acid exposure [Area under the curve (AUC)=0.597, p=0.179] and a marginally better and barely significant finding with total proximal acid exposure (AUC=0.651, p=0.036) in patients with aspiration of refluxate. The scintigraphic variables and IOM were comparatively better performers in the prediction of pulmonary aspiration of refluxate (AUC~0.850).

It must however be acknowledged that the published data which validates 24-hour pH monitoring is fundamentally concerned with the typical symptoms of heartburn and acid regurgitation. This imposes a significant limitation and may subsequently lead to an under diagnosis, particularly in the group of patients with silent reflux. In this group of patients, pH testing may not be the optimal choice of test for diagnosis of the disease. Some theories suggest that neutral or basic pH is equally or more significant than acidic pH. Refluxate may contain pepsin and bile contents that have also been implicated in tissue injury in the laryngopharynx (20,21). The data presented here illustrates the poor correlation between positive distal pH and pulmonary aspiration (3). Some studies have demonstrated pepsin in the laryngeal epithelium after a reflux event and questions have been raised as to the potential damage which may be caused (20,21). Failure to identify this group of patients could subsequently lead to progression of the disease and the development of secondary manifestations (22,23) such as laryngeal carcinoma, vocal cord granulomas and pulmonary aspiration and its multiplicity of complications such as bronchiectasis, lung fibrosis etc. The major diagnostic issue is attempting to imply the presence of refluxate through indirect markers of pH monitoring and manometry. Scintigraphic studies allow direct visualization of activity in the laryngopharynx and lungs. Importance of the diagnostic algorithm for LPR versus GORD is that LPR requires more stringent medical therapy, which has a high failure rate and leads to earlier contemplation of fundoplication, particularly if there is lung aspiration of refluxate (5).

The negative and positive predictive values of the scintigraphic time-activity curves for the oesophagus and pharynx as predictors of pulmonary aspiration were very good at 97% and 98%, respectively. This was an unexpected finding and may prove to be of clinical value in patients with a high clinical suspicion of aspiration, but no scan evidence in the delayed study. It may inform the decision to undertake fundoplication for severe cases of reflux with a strong clinical suspicion of aspiration. It is also reassuring to physically see GORD in the dynamic studies and then refluxate in the lungs in the delayed phase of the study, particularly in silent (heartburn negative) disease with manifest extra-oesophageal symptoms such as cough, globus etc.

The principal weakness of this study is the highly selected cohort of patients who already had a high pre-test probability of disease. It requires assessment in general community patients to ascertain its false positive rate. To this end, we have commenced a study in normal subjects with acquisition of reflux studies in 25 asymptomatic volunteers. Preliminary findings in 10 cases demonstrates low-grade gastroesophageal reflux in three and then to the mid-esophagous when in the upright position only. The others showed no evidence of reflux. There was no pharyngeal reflux or lung aspiration of tracer.

## Conclusion

We describe an innovative nuclear scintigraphic reflux test and its performance on a cohort of patients referred to a tertiary referral center for failure to respond to therapy of typical or atypical symptoms. This test has the potential to re-define the current understanding of GORD as it considers the broad definition of GORD. A strong correlation was found between scintigraphic parameters in the pharynx and upper oesophagus, and markers of ineffective oesophageal clearance consistent with dysmotility. These parameters were strongly predictive of pulmonary aspiration of the refluxate. pH studies were weakly correlated with these parameters and of little use in predicting laryngeal exposure and pulmonary aspiration.

## Figures and Tables

**Table 1:**
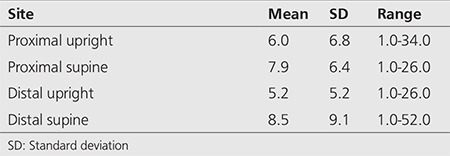
pH study (% acidic reflux/24 hours)

**Table 2:**
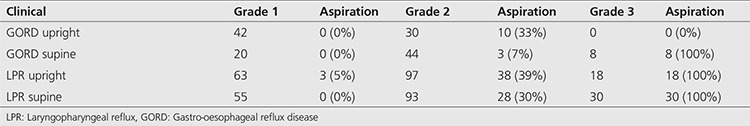
Pharyngeal time-activity curves for the scintigraphic studies according to symptom profile (laryngopharyngeal reflux versus gastro-oesophageal reflux disease)

**Table 3:**
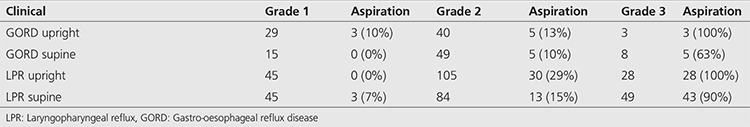
Upper oesophageal time-activity curves for the scintigraphic studies according to symptom profile (laryngopharyngeal reflux versus gastro-oesophageal reflux disease)

## References

[1] Spechler SJ (1992). Epidemiology and natural history of gastro-oesophageal reflux disease. Digestion.

[2] Vakil N, van Zanten SV, Kahrilas P, Dent J, Jones R;, Global Consensus Group (2006). The Montreal definition and classification of gastroesophageal reflux disease: a global evidence-based consensus. Am J Gastroenterol.

[3] Richter JE (2007). How to manage refractory GERD. Nat Clin Pract Gastroenterol Hepatol.

[4] De Giorgi F, Palmiero M, Esposito I, Mosca F, Cuomo R (2006). Pathophysiology of gastro-oesophageal reflux disease. Acta Otorhinolaryngol Ital.

[5] Koufman J, Aviv JE, Casiano RR, Shaw GY (2002). Laryngopharyngeal Reflux: Position Statement of the Committee on Speech, Voice, and Swallowing Disorders of the American Academy of Otolaryngology- Head and Neck Surgery. Otolaryngol Head Neck Surg.

[6] Caglar M, Volkan B, Alpar R (2003). Reliability of radionuclide gastroesophageal reflux studies using visual and time-activity curve analysis: inter-observer and intra-observer variation and description of minimum detectable reflux. Nucl Med Commun.

[7] Kjellen G, Brudin L, Hakansson HO (1991). Is scintigraphy of value in the diagnosis of gastrooesophageal reflux disease?. Scand J Gastroenterol.

[8] Shay SS, Abreu SH, Tsuchida A (1992). Scintigraphy in gastroesophageal reflux disease: a comparison to endoscopy, LESp, and 24-h pH score, as well as to simultaneous pH monitoring. Am J Gastroenterol.

[9] Falk GL, Beattie J, Ing A, Falk SE, Magee M, Burton L, Van der Wall H (2015). Scintigraphy in laryngopharyngeal and gastroesophageal reflux disease: a definitive diagnostic test?. World J Gastroenterol.

[10] Belafsky PC, Postma GN, Koufman JA (2002). Validity and reliability of the reflux symptom index (RSI). J Voice.

[11] Kahrilas PJ, Dodds WJ, Hogan WJ, Kern M, Arndorfer RC, Reece A (1986). Esophageal peristaltic dysfunction in peptic esophagitis. Gastroenterology.

[12] Kahrilas PJ, Dent J, Dodds WJ, Hogan WJ, Arndorfer RC (1987). A method for continuous monitoring of upper esophageal sphincter pressure. Dig Dis Sci.

[13] Ruth M, Carlsson S, Mansson I, Bengtsson U, Sandberg N (1993). Scintigraphic detection of gastro-pulmonary aspiration in patients with respiratory disorders. Clin Physiol.

[14] Barish CF, Wu WC, Castell DO (1985). Respiratory complications of gastroesophageal reflux. Arch Intern Med.

[15] Richter JE, Wu WC, Johns DN, Blackwell JN, Nelson JL, Castell DO (1987). Esophageal manometry in 95 healthy adult volunteers. Variability of pressures with age and frequency of “abnormal” contractions. Dig Dis Sci.

[16] Agreus L (1998). The epidemiology of functional gastrointestinal disorders. Eur J.

[17] Kastelik JA, Redington AE, Aziz I, Buckton GK, Smith CM, Dakkak M, Morice AH (2003). Abnormal oesophageal motility in patients with chronic cough. Thorax.

[18] Gastal OL, Castell JA, Castell DO (1994). Frequency and site of gastroesophageal reflux in patients with chest symptoms. Studies using proximal and distal pH monitoring. Chest.

[19] Charbel S, Khandwala F, Vaezi MF (2005). The role of esophageal pH monitoring in symptomatic patients on PPI therapy. Am J Gastroenterol.

[20] Gill G, Johnston N, Buda A, Pignatelli M, Pearson J, Dettmar PW, Koufman J (2005). Laryngeal epithelial defenses against laryngopharyngeal reflux: investigations of E-cadherin, carbonic anhydrase isoenzyme III, and pepsin. Ann Otol Rhinol Laryngol.

[21] Johnston N, Knight J, Dettmar PW, Lively MO, Koufman J (2004). Pepsin and carbonic anhydrase isoenzyme III as diagnostic markers for laryngopharyngeal reflux disease. Laryngoscope.

[22] Khan AM, Hashmi SR, Elahi F, Tariq M, Ingrams DR (2006). Laryngopharyngeal reflux: A literature review. Surgeon.

[23] Rathod NR (2010). Extra-oesophageal presentation of gastro-oesophageal reflux disease. J Indian Med Assoc.

